# Delayed Diagnosis of Congenital Heart Diseases in Adults: Opportunities for Improving Diagnostic Strategies

**DOI:** 10.3390/jcdd13090438

**Published:** 2026-09-06

**Authors:** Rea Levicki, Zrinko Pešut, Lora Levicki, Ivan Vukoja

**Affiliations:** 1 Department of Cardiology, Požega General Hospital, 34000 Požega, Croatia; 2 Department of Cardiology, General Hospital “Dr. Josip Benčević”, 35000 Slavonski Brod, Croatia; zrink0@yahoo.com; 3 Faculty of Medicine Osijek, “Josip Juraj Strossmayer” University of Osijek, 31000 Osijek, Croatia; levicki.lora@gmail.com

**Keywords:** congenital heart diseases in adults, first detection of congenital heart disease

## Abstract

(1) Background: In routine clinical practice, CHDs are occasionally diagnosed in adulthood, mostly relatively minor defects that become symptomatic later in life. Less frequently, adults may present with complex CHDs that, due to anatomical variations, remain undiagnosed until adulthood. Another important group comprises patients with previously unrecognized CHDs, particularly those from socioeconomically disadvantaged settings with limited access to healthcare. (2) Body: In adulthood, CHDs often present with nonspecific symptoms suggestive of other cardiovascular conditions (cardiomyopathy or coronary artery disease), including dyspnea, chest discomfort, signs of HF, syncope, palpitations, and paradoxical embolism. The initial manifestation may also be a malignant arrhythmia or sudden cardiac death; therefore, early recognition and diagnosis are crucial. TTE represents the first-line diagnostic modality. Reduced LVEF in young patients, particularly when accompanied by left atrial and right-sided cardiac chamber dilatation, should raise suspicion of an underlying CHD. The diagnostic evaluation may be supplemented by TEE, CMR imaging, and right heart catheterization. Familial forms of CHD and the risk of transmission to offspring have been extensively investigated, particularly for conditions with a strong genetic component, such as BAV. (3) Conclusions: In adult patients with newly diagnosed CHD, accurate recognition of clinical symptoms and completion of an appropriate diagnostic workup are essential for establishing the diagnosis and initiating timely treatment. Furthermore, identification of families at increased genetic risk is important to facilitate targeted genetic panel screening. Future research should focus on developing and validating appropriate screening strategies for the early detection of CHDs in resource-limited regions.

## 1. Introduction

In routine clinical practice, cardiologists frequently care for patients with congenital heart diseases (CHDs) who are transitioning from long-term pediatric follow-up to adult healthcare services, but also a subset of patients who are diagnosed with a CHD for the first time in adulthood. As a consequence of the prolonged asymptomatic or minimally symptomatic phase of the disease, particularly in cases involving smaller congenital defects, CHDs may remain undetected until adulthood, and they are often diagnosed only after the development of complications, such as heart failure, cardiac arrhythmias, right heart chamber dilatation, pulmonary hypertension or other related sequelae [1,2,3]. The most common CHDs encountered in adulthood can be broadly classified into two categories: acyanotic defects, including atrial septal defect (ASD), patent ductus arteriosus (PDA), and coarctation of the aorta (CA), and cyanotic defects, most notably tetralogy of Fallot (ToF). Other congenital cardiac conditions, although less frequently diagnosed in adulthood, include Ebstein’s anomaly, univentricular heart, and congenitally corrected transposition of the great arteries (ccTGA) [1].

For a better understanding of the pathophysiology and hemodynamic changes associated with unrepaired CHD in adulthood, it is important to recall the classification of CHDs according to anatomical location and the predominant hemodynamic characteristics: left-sided obstructive lesions including mitral valve (MV) stenosis, aortic valve stenosis, subaortic stenosis, supravalvular aortic stenosis, bicuspid aortic valve (BAV), and coarctation of the aorta; right-sided obstructive lesions including double-outlet right ventricle (RV), infundibular stenosis, pulmonary valve (PV) stenosis, supravalvular PV stenosis, and shunt lesions; and atrial septal defect (ASD) and ventricular septal defect (VSD), atrioventricular valve anomalies (MV anomalies and Mb Ebstein) and complex congenital heart diseases (ToF, ccTGA) including Fontan circulation [4]. Given the advances in cardiovascular diagnostics and the surgical management of CHD, an increasing number of children with CHD survive into adulthood. Consequently, the prevalence of CHDs, including both repaired and unrepaired forms, among adults has now exceeded that observed in the pediatric population [5]. Accordingly, data from the Finnish Population Registry suggest that the remarkable improvement in survival among patients with CHDs is largely attributable to advances in cardiac surgical techniques. These developments have led to substantial gains in long-term survival, even among individuals with complex congenital heart diseases. For instance, survival among patients with TGA reached 93% in 2009, compared with only 71% in the 1950s. Furthermore, ongoing progress in the diagnosis and management of CHDs has enabled corrective interventions to be performed at increasingly younger ages, contributing to a significant reduction in early mortality [6].

Delayed recognition of CHD during infancy and early childhood is known to adversely affect clinical outcomes and increase the risk of subsequent complications. In a prospective cohort study of 744 children under the age of 10 years, patients with a delayed diagnosis of CHD exhibited a significantly higher incidence of serious complications, such as cardiogenic shock, pulmonary hypertensive crises, and infective endocarditis [7]. The optimal window for the detection of CHDs is within the first 48–72 h after birth; diagnoses made after this period are typically considered delayed. While the majority of CHDs are identified during the neonatal period or early childhood, some patients with subtle clinical manifestations may escape detection until adulthood. Diagnosis in these individuals is frequently triggered by the progression of symptoms or the onset of late disease complications. For example, coarctation of the aorta (CA) represents one of the congenital cardiac anomalies most frequently identified later in life [8]. Among the CHDs that may remain undetected until adulthood, the most frequently encountered are ASD, VSD, CA, BAV, PDA, Ebstein anomaly, and partial anomalous pulmonary venous connection (PAPVC) [3,9,10,11,12]. Moreover, although exceedingly rare, complex congenital cardiac diseases, such as TGA, may be diagnosed in adulthood [13]. Considering the potential for congenital heart diseases to remain undiagnosed until adulthood or even middle age, it is important to evaluate patients who may be at risk for underlying CHD by assessing their clinical characteristics, together with pathological electrocardiographic and echocardiographic findings and, ultimately, relevant laboratory markers to facilitate timely referral to specialized CHD centers for comprehensive diagnostic evaluation, including transthoracic and transesophageal echocardiography, cardiac magnetic resonance imaging (MRI), myocardial scintigraphy, and other appropriate diagnostic modalities.

## 2. Materials and Methods

The review is based on a comprehensive literature search conducted in the PubMed, Embase, Cochrane Library, Scopus, and Web of Science databases. Searches were conducted using the following keywords: *congenital heart diseases in adulthood*, *late detection of congenital heart diseases in adulthood*, *congential heart diseases and atrial fibrillation*, *congenital heart diseases and pulmonary hypertension*, *congenital heart diseases and right ventricular dilatation*, *genetics of congenital heart diseases, natural history of congenital heart diseases,* and *artificial intelligence in the diagnosis and detection of congenital heart disease*. Inclusion criteria comprised studies published in English. Exclusion criteria included letters and non-peer-reviewed articles. The literature review encompasses studies published between Fall 2002 and June 2026, focusing on clinically relevant findings and their implications for the diagnosis of adult congenital heart diseases.

The structure of this review is as follows: Section 3: Clinical Presentation of Adults with First Diagnosed CHD; Section 4: Electrocardiographic Findings and Common Cardiac Arrhythmias in Adults with First Diagnosed Congenital Heart Disease; Section 5: Echocardiographic and other Imaging Findings in Adults with First Diagnosed Congenital Heart Disease; Section 6: Genetic Test Results in Adults with First Diagnosed CHD; Section 7: Discussion, Section 8: Conclusions.

## 3. Clinical Presentation of Adults with First Diagnosed CHD

Despite significant advances in pediatric cardiology and echocardiographic diagnostics, together with increased awareness of risk factors for CHD, a subset of CHDs remains undiagnosed until later in life, with diagnosis occasionally occurring in adulthood, sometimes including middle and older age [1,2]. The most common reason for delayed diagnosis is the presence of a relatively small congenital cardiac defect that does not produce clinically apparent manifestations over an extended period. Consequently, symptoms often emerge only in adulthood. However, the majority of symptoms reported by patients diagnosed with CHD in adulthood are relatively nonspecific, including exertional dyspnea, palpitations, anginal chest pain, and syncope. This nonspecific clinical presentation frequently contributes to missed or delayed diagnosis [14]. Particularly in older adults, the presentation of nonspecific symptoms such as exertional dyspnea or syncope are frequently attributed to other more prevalent age-related conditions, such as coronary artery disease (CAD) or the development of secondary cardiomyopathy [15,16]. Consequently, the possibility of an underlying CHD, is rarely considered.

One of the most common CHD first diagnosed in adulthood is an ASD. Its clinical presentation in adulthood varies considerably, ranging from small, clinically insignificant defects that may remain asymptomatic to rare and potentially serious manifestations, such as transient ischemic attack, acute limb ischemia, and paradoxical peripheral arterial embolism [17]. ASD and VSD are congenital shunt lesions that, when left undiagnosed until adulthood, typically present with biventricular heart failure, a significant left-to-right shunt leading to right ventricular dilatation and volume overload, and subsequent pulmonary hypertension [3,18]. Auscultatory findings in patients with ASD undetected until adulthood typically include a fixed split of the second heart sound (S2) or a systolic murmur over the pulmonary valve, whereas in patients with first diagnosed VSD, a holosystolic murmur is typically found [2]. Delayed diagnosis of an atrial septal defect or ventricular septal defect associated with a significant left-to-right shunt increases the likelihood of prolonged pulmonary hypertension and progressive elevation of pulmonary vascular resistance, ultimately leading to shunt reversal (right-to-left), cyanosis, and the development of Eisenmenger syndrome [2,19]. In addition to the chronic changes that may develop in patients with an uncorrected ASD or VSD, serious, although rare, acute complications may also occur. In patients without conventional risk factors for coronary artery disease who present with acute coronary syndrome, particularly when coronary lesions are suggestive of paradoxical embolism, comprehensive diagnostic evaluation, including transesophageal echocardiography, may identify a sinus venosus ASD with partial anomalous pulmonary venous return (PAPVR) as the underlying cause of paradoxical embolism [20].

Heart failure frequently represents the initial presentation of previously undiagnosed complex CHD in adulthood. The prevalence of heart failure is, in general, particularly high in those with cyanotic CHD (41%), Fontan circulation (30%), and a systemic right ventricle (25%) with known associated factors; age, atrial arrhythmias, infective endocarditis, pacemaker implantation, end-organ failure, higher NYHA functional class, increased heart rate, and pulmonary hypertension [20,21]. Accordingly, HF is common in adults with ToF, with the hemodynamic mechanisms underlying its development differing substantially between patients with unrepaired CHD and those who have undergone surgical correction [22]. In unrepaired ToF, right ventricular outflow tract (RVOT) obstruction leads to increased RV pressure, resulting in RV hypertrophy, impaired ventricular filling, and diastolic dysfunction, ultimately contributing to the progressive development of heart failure with preserved ejection fraction (HFpEF). However, in patients with repaired ToF, significant pulmonary regurgitation frequently develops later in life as a consequence of the surgical correction of pulmonary stenosis. This results in progressive RV volume overload and impaired RV systolic function, ultimately leading to the development of heart failure with reduced ejection fraction (HFrEF) [23].

Although ToF typically presents with cyanosis during the neonatal period, the clinical manifestations in a cohort of 25 adults with previously undiagnosed, unrepaired ToF varied considerably. Most patients had a large VSD and a high prevalence of combined infundibular and valvular pulmonary stenosis. Congestive HF was present in 33% of cyanotic patients and 38% of acyanotic patients. Among patients with cyanosis, a diastolic murmur was auscultated in two patients, and a holosystolic murmur at the lower left sternal border was detected in three patients; in addition, eight patients had a systolic ejection murmur, with the point of maximal intensity located at the second or third left intercostal space. Among patients without cyanosis, nine had a holosystolic murmur at the lower left sternal border, four had a systolic ejection murmur at the pulmonary area, eleven had a split second heart sound (S2), and four exhibited an accentuated pulmonary component of the second heart sound (P2). Notably, the majority of patients reported a reduction in symptoms during the second decade of life, while the absence of pulmonary hypervascularity was observed in 70% of acyanotic patients [24]. In some cases, CHDs may remain undiagnosed until advanced age, particularly in the presence of multiple comorbidities usually associated with HF. For example, patent ductus arteriosus was diagnosed at the age of 88 years in a patient with heart failure with preserved ejection fraction (HFpEF), arterial hypertension, atrial fibrillation, and diabetes mellitus, as an unexpectedly detected finding [25].

CHDs, regardless of whether they are diagnosed in adulthood or recognized during childhood, may initially present with numerous extracardiac complications. Consequently, these patients have a higher incidence of ischemic stroke, particularly those with cyanotic CHD, as well as paradoxical embolism in the presence of right-to-left shunts. The prevalence of intracranial aneurysms is also increased, especially among patients with CA and BAV. In addition, impaired pulmonary and renal function, as well as congestive hepatopathy secondary to HF, are commonly observed [26]. According to data from a retrospective cohort study involving more than 29,000 adults with CHD, the cumulative risk of ischemic stroke was 6.1% in women and 7.7% in men. The most important predictors of ischemic stroke were heart failure, diabetes mellitus, and a recent myocardial infarction [27].

Nonspecific symptoms, such as palpitations and supraventricular tachycardia, may be the initial manifestation of complex CHDs in adulthood, as exemplified by TGA. The condition in which the aorta arises from the right ventricle and the pulmonary artery originates from the left ventricle, resulting in complete separation of the systemic and pulmonary circulations, and is associated with an extremely high mortality rate if left uncorrected during the first year of life. Although survival into adulthood without surgical correction is rare, it is possible in the presence of an ASD or VSD, which permits the mixing of the blood of the systemic and pulmonary circulations and thereby maintains adequate systemic oxygenation [11,13,28]. Based on published case reports of adults with newly diagnosed d-TGA, the first clinical presentation most frequently consisted of acute HF associated with palpitations or documented atrial flutter [11,13]. In the first case, d-TGA was associated with severe pulmonary hypertension, ASD, VSD, and a patent ductus arteriosus. Physical examination revealed a grade 3/6 holosystolic murmur, with the point of maximal intensity at the lower left sternal border, an accentuated second heart sound (P2), and a grade 3/6 diastolic murmur with its point of maximal intensity in the second left intercostal space [13]. In the second case, d-TGA was associated with the presence of a large ASD with a bidirectional shunt. Cardiac auscultation revealed an accentuated second heart sound (S2), along with systolic and diastolic murmurs best heard at the second left intercostal space along the left sternal border [11].

Symptoms such as headache, epistaxis, dizziness, tinnitus, intermittent claudication, and cold feet may be the initial manifestation of the CoA in adults with newly diagnosed arterial hypertension, particularly in those with milder forms of the condition [29]. In very rare cases, interrupted aortic arch (IAA) may also be diagnosed in adulthood. For example, the initial presentation may involve a patient with poorly controlled arterial hypertension, without a documented blood pressure difference between the upper and lower extremities, owing to the presence of extensive collateral circulation and a discontinuous aortic arch distal to the left subclavian artery [9]. Furthermore, congenital coronary artery anomalies, including the exceedingly rare condition of left main coronary artery ostial atresia supplied by retrograde flow from a dominant right coronary artery, may be diagnosed in young adulthood, presenting clinically as acute HF in secondary cardiomyopathy with reduced ejection fraction (HFrEF) [30].

Although BAV is one of the most common congenital heart defects (CHDs), with a prevalence of 0.5–2%, it frequently remains asymptomatic during childhood, with only 1 in 50 children developing clinically symptomatic BAV by adolescence [31,32]. BAV is frequently associated with concomitant involvement of the aortic root, and the clinical presentation in patients with previously undiagnosed BAV who reach adulthood depends on the type and severity of the underlying pathology. These complications may include the development of aortic stenosis, aortic regurgitation, or aortopathy characterized by dilatation of the ascending aorta, which, in the most severe cases, may progress to aortic dissection. Patients with an anatomically abnormal aortic valve are also at increased risk of infective endocarditis [32]. In patients with normal or mildly impaired function of the bicuspid aortic valve in young adulthood, with aging and progression of atherosclerosis, calcium accumulation in the aortic valve and annulus occurs, as well as dilatation of the ascending aorta, and almost in half of these patients, the development of late complications of the disease is observed, usually in the fourth and fifth decades of life, which requires further surgical treatment [33].

Although Ebstein anomaly, resulting from the apical displacement of the tricuspid valve and the consequent dilatation and dysfunction of the right ventricle, is most commonly diagnosed during the neonatal period due to cyanosis and right-sided heart failure, milder forms of the disease are often encountered in adults with previously undiagnosed Ebstein anomaly. In these patients, the initial presentation may include mild dyspnea or palpitations associated with supraventricular arrhythmias, owing to the frequent coexistence of an ASD and accessory conduction pathways, or atrial fibrillation [34,35]. Although cone repair has been shown to be effective in reducing tricuspid regurgitation and decreasing right ventricular size in patients with Ebstein’s anomaly when performed during either childhood or adulthood, delayed diagnosis in older patients, particularly after substantial dilatation of the right-sided cardiac chambers has occurred, with subsequent thrombus formation within the right atrium, is associated with increased perioperative morbidity during surgical correction of the defect [35,36].

## 4. Electrocardiographic Findings and Common Cardiac Arrhythmias in Adults with First Diagnosed Congenital Heart Disease

A population-based study involving more than 11,000 adults with CHDs investigated the most common cardiac rhythm abnormalities in this population, including tachyarrhythmias (8.7%), conduction disorders (1.5%), and combinations of these conditions (0.5%). Conduction disorders were more frequent among patients with more than one congenital heart defect. In addition, a higher proportion of patients with both conduction disorders and tachyarrhythmias had complex CHD (16.7%). Supraventricular arrhythmias were more common in patients with ASD, whereas ventricular arrhythmias occurred more frequently in patients with complex CHD, such as ToF, and in those with VSD [37]. The substrate for arrhythmogenesis in CHD may result from abnormal cardiac anatomy, including malposition of components of the cardiac conduction system (e.g., the sinoatrial and atrioventricular nodes), postoperative scarring following previous cardiac surgery, and genetically determined factors [38,39].

Malposition of the sinoatrial (SA) node may occur in left atrial isomerism, where the SA node may be absent, whereas in right atrial isomerism, two SA nodes may be identified [40]. In addition to patients with left atrial isomerism, complete atrioventricular (AV) block is frequently encountered in patients with TGA. Overall, patients with both corrected and uncorrected forms of TGA exhibited a high incidence of cardiac implantable electronic device therapy, with permanent pacemaker implantation required in 19% of cases, primarily for complete AV block, sick sinus syndrome, or slow atrial fibrillation, while implantable cardioverter–defibrillator (ICD) implantation was performed in 3% of patients [39,41].

Accessory atrioventricular pathways are characteristically associated with Ebstein’s anomaly, ccTGA, single-ventricle physiology, and atrioventricular septal defects [40]. Atrioventricular reciprocating tachycardia (AVRT) mediated by twin AV nodes, in which one AV node serves as the anterograde conduction pathway and the other as the retrograde conduction pathway, is a characteristic electrophysiological finding in complex CHDs involving major AV septal canal defects [42]. Monomorphic ventricular tachycardia (VT) is more commonly associated with the primary CHD, arising from an arrhythmogenic substrate related to abnormal cardiac anatomy, as observed in VSDs, ToF, tetralogy of Fallot with pulmonary atresia, and double-outlet right or left ventricle. In contrast, polymorphic VT is more frequently encountered in complex CHD, where the arrhythmogenic substrate is attributable not only to the underlying congenital anatomical abnormalities but also to postoperative scar tissue, as seen in congenitally corrected transposition of the great arteries (ccTGA) with VSD and Ebstein’s anomaly. Ventricular fibrillation has been reported in patients with dextro-transposition of the great arteries (d-TGA) ccTGA, and congenital aortic stenosis [43].

Atrial fibrillation may represent the initial clinical manifestation of adult CHD, occurring in both relatively simple defects, such as an ASD associated with cor triatriatum, and more complex lesions, including Ebstein’s anomaly diagnosed later in adulthood. In most cases, the initial presentation of CHD with atrial fibrillation is accompanied by early signs and symptoms of heart failure [35,44]. Atrial fibrillation and atrial flutter occur more frequently in older patients with ASD than during childhood [45]. According to a Danish registry study involving 151 adults with small, unrepaired atrial septal defects, who underwent 48 h Holter ECG monitoring, with patients in whom atrial fibrillation was detected subsequently undergoing 7-day Holter electrocardiography (ECG) monitoring, the incidence of arrhythmias remained high despite spontaneous closure of the defect in approximately 80% of patients. The most frequently observed arrhythmias were supraventricular tachycardia, non-sustained atrial arrhythmias, and atrial fibrillation [46]. It has been demonstrated that the incidence of supraventricular arrhythmias, particularly atrial flutter, decreases following ASD closure performed in adulthood (mean age at repair: 42 years). However, the incidence of atrial fibrillation remains unchanged after late ASD closure in this patient population [47].

Right bundle branch block (RBBB) was frequently observed in patients with CHDs. A small prospective study reported a high prevalence of interatrial septal abnormalities in patients with RBBB, identified in 80.5% of cases. These abnormalities included patent foramen ovale (PFO) in 39.0% of patients, atrial septal aneurysm in 21.9%, and ASD in 19.5% [48]. Although the diagnostic utility of ECG for identifying specific CHDs is relatively limited in both childhood and adulthood, several electrocardiographic features remain characteristic of particular congenital cardiac lesions. In addition to incomplete RBBB, a study involving 45 patients with secundum ASD demonstrated that the amplitude of the P wave in lead V3 correlated with the likelihood of an ASD measuring more than 5 mm in diameter [49]. In a cohort of 25 patients diagnosed with ToF in adulthood, the electrocardiogram demonstrated RBBB in 58% of cases. Tall, peaked P waves were observed in 66% of cyanotic patients, whereas predominant right ventricular hypertrophy was present in 46% of acyanotic patients [23]. In patients with Ebstein’s anomaly, characteristic electrocardiographic findings include tall P waves, prolonged PR interval, RBBB, and fragmented QRS complexes. In addition, owing to the frequent presence of multiple accessory atrioventricular pathways, Wolff–Parkinson–White (WPW) syndrome is commonly observed [50,51]. In a large cohort of pediatric and adult patients with CHD and HFrEF, characteristic electrocardiographic findings included abnormalities of the precordial QRS complexes and T waves. High-risk ECG features most commonly comprised deep S waves in lead V2 and T-wave inversion in the lateral precordial leads [52]. For the majority of CHDs diagnosed later in life, no specific ECG features have been identified. In a subset of patients, however, atrial fibrillation may represent the only electrocardiographic abnormality [18,25].

## 5. Echocardiographic and Other Imaging Findings in Adults with First Diagnosed Congenital Heart Disease

Echocardiography, including M-mode, two-dimensional (2D) imaging, three-dimensional (3D) and four-dimensional (4D) imaging, Doppler imaging, and the assessment of global longitudinal strain (GLS), is the gold standard for the diagnosis of CHDs in adults [29,53]. To complement the diagnostic evaluation, transoesophageal echocardiography, often incorporating 3D or 4D imaging modalities, is frequently required for the assessment of CHDs [29]. One of the principal clinical manifestations of CHD in adulthood is heart failure. As these patients frequently present initially to the emergency department, point-of-care echocardiographic findings that should raise suspicion of underlying CHD include cardiomyopathy with HFrEF in younger patients without identifiable secondary causes, particularly when accompanied by significant left atrial (LA) dilatation. Additional echocardiographic features suggestive of CHD include right heart chamber enlargement, characterised by RV basal dilatation (≥42 mm), RV hypertrophy (wall thickness ≥ 5 mm), and the presence of pulmonary hypertension. Patients presenting with these findings should be referred for comprehensive transthoracic echocardiography and, when clinically indicated, transoesophageal echocardiography to establish the underlying diagnosis [54,55].

The most common CHD that remains undiagnosed until adulthood, and is often detected incidentally during echocardiographic evaluation, is an ASD. The majority of ASDs are of the ostium secundum type (80%), characterised by a defect in the region of the fossa ovalis. Less frequently, ostium primum defects (15%) occur as part of atrioventricular septal defects located near to the mitral and tricuspid valves and are commonly associated with chromosome 21 trisomy. Other less common subtypes include sinus venosus defects located near the junction of the superior or inferior vena cava with the right atrium (approximately 5–6% of cases), whereas an unroofed coronary sinus is the rarest form, accounting for fewer than 1% of ASDs [56,57,58,59]. Depending on the type and size of the ASD, shunt direction, and RV volume overload, transthoracic echocardiography alone is often insufficient for comprehensive evaluation. In a smaller cohort study involving 154 patients with unrepaired atrial septal defects the sensitivity of TTE for the detection of ostium secundum atrial septal defects was 89%, compared with 100% for ostium primum ASD and 44% for sinus venosus defects. The specificity was 11% for ostium secundum ASD and 56% for sinus venosus defects [60]. Additional diagnostic imaging modalities, including transesophageal echocardiography (TEE), three-dimensional transesophageal echocardiography (3D TEE), cardiac magnetic resonance (CMR) imaging, cardiac computed tomography (CCT), and cardiac catheterization, are frequently required to ensure accurate diagnosis and appropriate assessment [61]. In adult patients with previously undiagnosed ASD, the initial clue to the diagnosis is often the detection of right ventricular (RV) dilatation, typically accompanied by preserved or hyperdynamic RV systolic function [29,62]. TEE is used to provide a more accurate diagnosis of sinus venosus defects and is also essential for the detailed assessment of secundum ASDs. Three-dimensional transesophageal echocardiography (3D TEE) further enables comprehensive visualization of the defect morphology, providing detailed information on its size, shape, and surrounding rims [29]. Contrast echocardiography is a useful modality for the detection of small intracardiac shunts, particularly in patients with suboptimal echocardiographic acoustic windows [62]. CMR for the assessment of right ventricular function and the pulmonary-to-systemic blood flow ratio (Qp:Qs > 1.5), together with cardiac catheterization in patients with a systolic pulmonary artery pressure (sPAP) > 40 mmHg, pulmonary vascular resistance (PVR) < 5 WU, are essential for guiding decisions regarding ASD closure in adult patients [29,62].

Based on the anatomical location of the interventricular septal defect, four types of VSDs are distinguished: perimembranous VSD (approximately 80%), which have a relatively high likelihood of spontaneous closure; muscular (trabecular) VSD (10–15%), which may be multiple and also frequently closes spontaneously; outlet VSD (approximately 5%), located beneath the semilunar valves (outlet septum), which are associated with aortic regurgitation (AR) due to prolapse of the right coronary aortic cusp and may also be accompanied by aneurysm of the sinus of Valsalva; and inlet VSD, located immediately beneath the atrioventricular (AV) valves and typically associated with atrioventricular septal defects (AVSD) [29]. Transthoracic echocardiography (TTE) is the first-line imaging modality for the detection of VSDs. In the parasternal long-axis view, perimembranous VSDs and a proportion of muscular VSDs can be visualized. The parasternal short-axis view at the level of the great arteries allows visualization of perimembranous and juxta-arterial (outlet) VSDs, whereas the parasternal short-axis view at the ventricular level is optimal for identifying muscular VSDs. Inlet VSDs are best demonstrated in the apical four-chamber view, while outlet VSDs are most clearly visualized in the subcostal (subxiphoid) view [63]. Colour Doppler and continuous-wave Doppler imaging are used to assess the continuity of the interventricular septum. Once a VSD is identified, the peak pressure gradient across the defect should be measured. A peak pressure gradient > 64 mmHg is suggestive of a restrictive VSD, whereas values between 25 and 60 mmHg indicate a moderately restrictive defect [64]. Moreover, 3D TTE and GLS are also used for the adequate assessment of left ventricular size and function, with 3D TTE enabling volumetric assessment. TEE is particularly useful for detailed visualization of the AV valves, the perimembranous portion of the interventricular septum (IVS), inlet VSDs, and the morphology of the aortic valve cusps, particularly for identifying prolapse of the right coronary cusp [63]. CMR serves as a complementary imaging modality to TTE and TEE for the assessment of shunt size, while cardiac catheterization is performed in patients with elevated systolic pulmonary artery pressure (sPAP) to determine pulmonary vascular resistance [29]. In patients with an unrecognized VSD and high trans-VSD pressure gradients, the development of a double-chambered right ventricle (DCRV) may occur as a late complication. It is often asymptomatic during childhood, with the first clinical manifestations and diagnosis frequently occurring in adulthood. This abnormality may be overlooked on TTE, making TEE and CMR necessary for accurate detection [12,65].

Ebstein’s anomaly is primarily characterized by a malformation of the tricuspid valve, in which the septal and posterior leaflets are adherent to the underlying myocardium, while the anterior leaflet is typically redundant and fenestrated. The anomaly is further characterized by apical displacement of the functional tricuspid annulus, atrialization of the right ventricle, and dilatation of the right atrioventricular junction (true tricuspid annulus). The point of maximal apical displacement of the septal and posterior tricuspid valve leaflets relative to the anterior leaflet is located at the commissure between the septal and posterior leaflets. In Ebstein’s anomaly, the displacement at this level ex-ceeds 8 mm/m^2^ of body surface area (BSA), which represents a diagnostic criterion for the condition [66,67]. The altered cardiac anatomy in Ebstein’s anomaly leads to progressive dilatation of the right-sided cardiac chambers, most frequently in association with significant tricuspid regurgitation [68]. The displaced tricuspid valve divides the right ventricle into two distinct components: the inlet portion, which represents the functional atrialized segment of the right ventricle, and the remaining functional right ventricle, which consists of the trabecular and outlet portions [69]. Mitral valve prolapse is a common finding in patients with Ebstein’s anomaly, likely related to compression and geometric distortion of the left ventricle [68]. The diagnosis of Ebstein’s anomaly is established in the majority of cases at birth or during early childhood. However, in a subset of oligosymptomatic or asymptomatic patients, the diagnosis may not be established until adulthood or even later in life. Adult patients diagnosed with Ebstein’s anomaly are less likely to have a concomitant ASD, which may contribute to a more benign clinical course and delayed symptom onset [10]. The diagnosis of Ebstein’s anomaly can usually be established readily by TTE. In addition to the rapid visualization of dilated right-sided cardiac chambers and the dilated atrialized portion of the RV, TTE enables identification of the apical displacement of the septal and posterior tricuspid valve leaflets, best assessed in the apical four-chamber and right ventricular inflow views. Doppler echocardiography further allows assessment of the severity of tricuspid regurgitation, which is commonly significant [70,71]. TEE provides superior visualization of the tricuspid valve and annulus, particularly in the mid-esophageal four-chamber and transgastric sagittal views. It is also valuable for confirming the presence of a concomitant ASD or a patent foramen ovale (PFO) [71]. In adult patients with unrepaired Ebstein’s anomaly, CMR has proven to be a valuable imaging modality for the assessment of right heart function. CMR enables accurate volumetric analysis using short-axis and axial views to quantify the functional right ventricular end-diastolic volume (RVEDV) and end-systolic volume (RVESV) [72].

Tetralogy of Fallot is defined by the presence of four characteristic features: a ventricular septal defect (VSD), overriding of the aorta, right ventricular hypertrophy, and right ventricular outflow tract (RVOT) obstruction [73]. The diagnosis of tetralogy of Fallot is usually established shortly after birth due to the presence of cyanosis. In patients with unrepaired tetralogy of Fallot, mortality approaches 50% within the first few years of life, while survival beyond 30 years of age is exceedingly rare without surgical correction [74]. The VSD in ToF results from the cranial displacement of the infundibular (outlet) septum. Consequently, the infundibular septum either fails to align and fuse properly with the muscular interventricular septum during development or fails to undergo muscularization and remains fibrous. In both cases, its cranial malalignment results in the characteristic VSD. Because of the displacement of the malaligned infundibular (outlet) septum toward the RV, the aortic root overrides the muscular interventricular septum. When significant right ventricular outflow tract obstruction is present, the VSD predominantly shunts blood from right to left, resulting in cyanosis. With significant aortic override, the morphology approaches that of a double-outlet right ventricle (DORV) [75]. Given the characteristic clinical presentation, the high prevalence of cyanosis in patients with ToF, and distinctive features such as digital clubbing and a characteristic pansystolic murmur, most cases are diagnosed during childhood. However, in rare instances, particularly in resource-limited settings with restricted access to healthcare, the initial presentation may occur in adulthood. Delayed diagnosis may also be seen in acyanotic patients with milder degrees of pulmonary stenosis. The diagnosis is confirmed by TEE, which allows comprehensive assessment of the cardiac anatomy, including the VSD, the degree of aortic override, the severity of RVOT obstruction, and RV hypertrophy [76]. In two reported cases of patients in whom ToF was diagnosed in the fourth and fifth decades of life, respectively, TTE demonstrated the characteristic anatomical features of the defect. In the first patient, the findings included a large malalignment subaortic VSD, an overriding aorta of less than 50%, consistent with abnormal conal septal development, RV hypertrophy, infundibular pulmonary stenosis, and an interatrial septal aneurysm. In the second patient, echocardiography revealed a large subaortic VSD, an overriding aorta, severe valvular pulmonary stenosis, and marked RV hypertrophy [76,77]. On chest radiography, ToF was classically characterized by a boot-shaped cardiac silhouette [75].

The clinical presentation of patients with patent ductus arteriosus (PDA) varies considerably depending on the size of the defect, the degree of left ventricular volume overload, and the development of pulmonary hypertension, which results in pressure overload of the right ventricle. In rare cases, disease progression may ultimately lead to the development of Eisenmenger syndrome. Most patients with an established diagnosis have a relatively mild form of the defect [29]. In approximately 14% of patients with patent ductus arteriosus (PDA), calcification of the ductus can be detected on chest radiography [78]. Transthoracic echocardiography (TTE) is used to visualize the PDA and assess left ventricular volume overload, estimate systolic pulmonary artery pressure (sPAP), evaluate the size of the pulmonary artery, and assess right heart chamber dimensions and function. The magnitude of the left-to-right shunt (Qp) is quantified by cardiovascular magnetic resonance (CMR), whereas in patients with elevated sPAP, right heart catheterization is performed to determine pulmonary vascular resistance (PVR) [29].

In adults CA is primarily evaluated by TTE, which can raise suspicion of the diagnosis but is often insufficient to establish a definitive. Therefore, pulsed-wave (PW) Doppler assessment of flow in the abdominal aorta is an important diagnostic adjunct. A flow pattern characterized by reduced pulsatility, a low systolic peak velocity, and the absence of early diastolic flow reversal is highly suggestive of coarctation of the aorta [79]. Using the suprasternal transthoracic echocardiographic view, the aortic arch can be visualized, while Doppler imaging demonstrates turbulent flow at the site of the coarctation with an increased peak pressure gradient across the narrowed aortic segment [80]. In adults with coarctation of the aorta (CoA), global longitudinal strain (GLS) and left atrial strain can detect early impairment of myocardial function before a decline in left ventricular ejection fraction (LVEF) or significant left atrial enlargement becomes apparent. Interestingly, following CoA repair, GLS values often remain reduced despite preservation of LVEF, suggesting persistent subclinical left ventricular systolic dysfunction [81,82]. Cardiovascular magnetic resonance (CMR) and cardiac computed tomography (CT) with three-dimensional (3D) reconstruction are the gold standard for comprehensive evaluation of the entire thoracic aorta and for establishing the diagnosis of coarctation of the aorta (CoA). These imaging modalities are also essential for the long-term surveillance of patients after CoA repair, enabling the detection of restenosis, aneurysm formation, and other postoperative complications [80].

Bicuspid aortic valve represents a spectrum of conditions depending on morphology (number of cusps, commissures, number of raphes, condition of the ascending aorta) and function (predominantly stenosis, predominantly regurgitation, or a balance between the two). The so-called “true” BAV refers to two completely developed cusps, 0 raphes, and is a less common form. More commonly, there are three formed cusps with 1–2 raphes. In the case of 1 raphe, the non-coronary cusp is usually larger, while the right and left coronary cusps are incompletely separated, smaller, and malformed. In the case of 2 raphes, they are usually located between the right and left coronary cusps and between the right coronary and non-coronary cusps, and this form is associated with the highest incidence of aneurysm of the ascending aorta [82,83]. The diagnosis of BAV is primarily established by TTE, with high sensitivity (92%) and specificity (96%), where adequate visualization of the aortic valve in systole and diastole in the parasternal short-axis view is required, in which the characteristic “fish-mouth” opening of the valve can be seen, while “doming” of the aortic cusps is a characteristic finding in the parasternal long-axis view [84]. TEE is used for a more detailed visualization of the aortic cusps in cases of an inadequate TTE window, while MSCT is used to assess the dimensions of the ascending aorta [32].

## 6. Genetic Test Results in Adults with First Diagnosed Congenital Heart Disease

Patients with congenital heart disease (CHD) frequently exhibit chromosomal abnormalities or aneuploidies (8–12%), copy number variations (3–25%), and pathogenic single-gene variants (3–5%). The likelihood of identifying a genetic cause of CHD is higher in patients with nonisolated CHD than in those with an isolated cardiac defect [85]. In a large cohort of more than 2800 patients with CHD, “de novo” mutations were identified in 8% of cases. Their prevalence was 3% among patients with isolated CHD and 28% among those with CHD accompanied by extracardiac anomalies [86]. One of the most common examples of aneuploidy is trisomy 21 (Down syndrome), which is most frequently associated with congenital heart defects involving the “crux cordis”, including ASD, VSD, and AVSD [85,87]. Turner syndrome is characterized by complete or partial loss of one X chromosome. It is associated with a broad spectrum of cardiovascular abnormalities, including hypertension, cardiac conduction disorders, mitral valve prolapse, and coarctation of the aorta, often accompanied by a BAV. In addition, patients with Turner syndrome have a markedly increased risk of aortic dilatation and aortic dissection compared with the general population [88]. CHD frequently occur as part of genetic syndromes. For example, ToF is commonly associated with Turner syndrome, DiGeorge syndrome (22q11.2 deletion syndrome), Down syndrome, and Williams–Beuren syndrome [26,73].

Table 1 below summarizes a group of genes with established roles in the pathogenesis of CHD. These genes predominantly encode transcription factors, signaling molecules, and structural proteins that are essential for cardiac development, morphogenesis, and normal cardiac function.

In patients with familial CHD, mutations in NKX2-5 have been strongly associated with ASD and atrioventricular (AV) block. According to published data, this phenotype is observed in approximately 74% of affected individuals, while approximately 15% of patients with both ASD and AV block experience sudden cardiac death [89]. The G296S missense mutation in GATA4, another key cardiac transcription factor, results in reduced transcriptional activity of GATA4 and disrupts its interaction with TBX5, thereby contributing to the development of septal defects. Similar pathogenic mechanisms have also been demonstrated for other GATA4 mutations [90]. Mutations affecting the NOTCH1 signaling pathway influence multiple cellular processes and play a pivotal role in aortic valve development. NOTCH1 variants have a significant impact on the aortic valve phenotype in adults with familial BAV, promoting calcification of the bicuspid valve and the subsequent development of aortic stenosis. A very high frequency of inheritance of BAV, as well as other associated cardiovascular malformations, is well known. In a study conducted in more than 300 members of three generations of families of patients with known BAV, a prevalence of 31% of BAV and/or associated cardiovascular malformations (ASD, VSD, mitral valve disease, dilatation of the ascending aorta) was demonstrated [91]. Other study conducted in 38 families (353 subjects) with a known history of BAV and/or associated cardiovascular malformation identified regions of chromosomes 18q, 5q, and 13q responsible for the development of the disease [92]. Furthermore, impaired structural integrity of the aortic wall associated with NOTCH1 dysfunction may contribute to aortic root dilation and secondary aortic regurgitation [93]. Mutations in MYH6 and MYH7 have been associated with a wide spectrum of congenital heart defects, including VSD, ASD, and left ventricular outflow tract (LVOT) obstruction. In a cohort of patients with familial left ventricular noncompaction (LVNC), a “de novo” mutation in the MYH7 gene, encoding the β-myosin heavy chain, was identified and shown to segregate with the disease in several affected family members. The same mutation was also associated with Ebstein anomaly and ASD [94].

According to the Danish nationwide registry study including 18,708 patients with CHD, the recurrence risk ratio among first-degree relatives varied substantially according to the specific cardiac phenotype. The highest recurrence risk was observed for heterotaxy (risk ratio [RR] 79.1), followed by RVOT obstruction (RR 48.6), AVSD (RR 24.3), LVOT obstruction (RR 12.9), conotruncal defects (RR 11.7), isolated ASD (RR 7.1), and isolated VSD (RR 3.4). Overall, individual CHD phenotypes demonstrated marked but highly variable familial clustering among first-degree relatives, with recurrence risks ranging from approximately threefold to 80-fold higher than in the general population. In contrast, the risk of discordant (phenotypically dissimilar) CHD among first-degree relatives was considerably lower [95].

## 7. Discussion

Although the majority of CHD are diagnosed during childhood, a proportion of patients remain undiagnosed during this period, most commonly due to the asymptomatic or oligosymptomatic nature of the disease at that time. Consequently, the initial diagnosis of CHD is often established only when disease-related complications develop, including heart failure, arrhythmias, right-sided cardiac chamber dilatation, and pulmonary hypertension [1,2,3]. Patients have a substantially more favorable clinical course, with a lower likelihood of late complications (such as cardiogenic shock, pulmonary hypertensive crises, and infective endocarditis) and ultimately improved long-term survival, when CHD requiring intervention based on clinical indications is surgically corrected [6,7]. The most common CHDs which may occasionally remain undiagnosed until adulthood include ASD, VSD, CA, BAV, PDA, Ebstein anomaly, and PAPVC. Rarely, more complex CHDs such as ToF and TGA may be diagnosed in adulthood, particularly in the presence of collateral shunt [3,10,11,12]. Given the known unfavorable outcomes associated with delayed correction of CHD that remain undiagnosed until adulthood, timely detection is of crucial importance. It is essential to identify patients at increased risk of underlying CHD based on their clinical presentation, physical examination findings, or initial TTE results. These patients should undergo an appropriate diagnostic evaluation, including complementary imaging modalities such as TEE, CMR, and cardiac catheterization, when indicated. Early establishment of the diagnosis enables appropriate clinical decision-making and timely initiation of further management.

Among patients with late-diagnosed CHD presenting in adulthood or advanced age, those who remained asymptomatic for an extended period most commonly had less severe structural defects. The first clinical presentation of CHD in adulthood is often nonspecific, including exertional dyspnea, palpitations, anginal chest pain, and syncope. Therefore, these symptoms can easily be misinterpreted as manifestations of other, more prevalent conditions affecting patients in this age group [14]. ASD and VSD that remain undiagnosed until adulthood typically present with signs of RV volume overload, the development of pulmonary hypertension, and progressive biventricular heart failure as a consequence of a left-to-right (L–R) shunt [3,18]. In cases of a significant shunt and a prolonged period of undiagnosed CHD, progressive worsening of pulmonary hypertension and an increase in pulmonary vascular resistance may occur, eventually leading to shunt reversal from right-to-left (R–L) and the development of Eisenmenger syndrome [2,19]. Complex CHD presenting in adulthood are most commonly associated with clinical signs of heart failure. In complex lesions such as ToF, the mechanisms underlying the development of heart failure differ substantially between repaired (HFrEF) and unrepaired patients (HFpEF) [23]. Patients presenting with tetralogy of Fallot (ToF) in adulthood generally represent a milder disease spectrum, most commonly characterized by pulmonary stenosis rather than more severe forms such as pulmonary atresia or complete pulmonary valve obstruction [24]. Cases of newly diagnosed dextro-transposition of the great arteries (d-TGA) in adulthood have been described, presenting with acute heart failure accompanied by supraventricular arrhythmias. However, these patients had a large ASD that functioned as a bidirectional shunt, allowing sufficient mixing of systemic and pulmonary blood flow [11,13]. CA is occasionally identified as an incidental finding during the evaluation of adults with arterial hypertension. Patients commonly present with symptoms related to upper-body hypertension, including headache, epistaxis, dizziness, and tinnitus [29]. Ebstein anomaly, characterized by apical displacement of the tricuspid valve and progressive severe tricuspid regurgitation, typically presents with signs and symptoms of heart failure. In adults with previously undiagnosed Ebstein anomaly, an associated ASD is frequently identified. In addition, owing to the high prevalence of accessory atrioventricular pathways, the initial clinical presentation may be tachyarrhythmia associated with Wolff–Parkinson–White syndrome [34,35]. In patients with BAV, symptoms may develop during the fourth or fifth decade of life due to the progression of atherosclerotic changes in the abnormal valve and calcium deposition within the wall of the ascending aorta, which may contribute to progressive dilatation of the ascending aorta [33].

The most common arrhythmias in patients with previously undiagnosed CHD presenting in adulthood are tachyarrhythmias, conduction disturbances, or a combination of both, particularly in patients with more complex CHDs. In these patients, malignant ventricular arrhythmias may also occur, especially in conditions such as ToF [36]. The arrhythmogenic substrate in adults with previously undiagnosed CHD is primarily related to abnormal cardiac anatomy, including malposition or even duplication of components of the cardiac conduction system, such as the SA and AV nodes. In addition, genetically determined factors may also contribute to the development of arrhythmias [38,39]. Atrial fibrillation is a common initial clinical presentation in adults with previously undiagnosed CHD and is frequently accompanied by signs and symptoms of heart failure. Congenital cardiac lesions in which atrial fibrillation commonly represents the initial manifestation include cor triatriatum and more complex defects, particularly Ebstein anomaly diagnosed later in adulthood [33,44]. The ECG may be entirely normal in many adults with previously undiagnosed CHD particularly in the absence of right-sided heart failure. Alternatively, the initial ECG may demonstrate a supraventricular tachyarrhythmia. However, certain CHDs, including secundum ASD, ToF, and Ebstein anomaly, are associated with characteristic electrocardiographic findings. These include tall P waves, PR interval prolongation, and RBBB, whereas fragmented QRS complexes are particularly characteristic of Ebstein anomaly [50,51,52]. In patients with CHD, prolonged Holter ECG monitoring may be warranted because of the higher likelihood of arrhythmias compared with the general population. A 48 h Holter ECG recording may therefore be appropriate, whereas a 7-day Holter ECG monitoring period may be preferable in patients with atrial fibrillation [46].

In adults with previously undiagnosed CHD presenting to the emergency department, focused TTE may reveal findings that should raise suspicion of an underlying congenital cardiac lesion. These include cardiomyopathy with HFrEF in younger patients without an identifiable secondary cause, particularly when accompanied by significant left atrial LA dilatation. Additional suggestive findings include right heart chamber enlargement, characterized by RV basal dilatation (≥42 mm), RV hypertrophy (wall thickness ≥ 5 mm), and the presence of pulmonary hypertension [54,55]. TTE is the primary imaging modality for the detection of CHD in adults. However, additional diagnostic investigations are frequently required to establish an accurate diagnosis and provide comprehensive anatomical and hemodynamic assessment. It is important to emphasize that TTE, as the first-line diagnostic modality, does not have uniform sensitivity and specificity for different types of atrial septal defects. In particular, its sensitivity for detecting sinus venosus ASD is as low as 44%. Therefore, in such cases, and preferably in other types of ASDs as well, TTE should be supplemented with transesophageal echocardiography (TEE). Other methods for detection are three-dimensional transesophageal echocardiography (3D TEE), cardiac magnetic resonance (CMR) imaging, cardiac computed tomography (CCT), and cardiac catheterization [62]. For example, in the evaluation of adults with ASD for potential defect closure, cardiac magnetic resonance (CMR) imaging is used to assess right ventricular function and quantify the pulmonary-to-systemic blood flow ratio (Qp:Qs > 1.5). In patients with elevated systolic pulmonary artery pressure (sPAP > 40 mmHg), right heart catheterization is required to determine pulmonary vascular resistance (PVR). A PVR < 5 Wood units, together with the overall clinical and hemodynamic assessment, is an important criterion supporting the decision to proceed with ASD closure in adult patients [29,62]. Echocardiographic findings vary according to the specific type of CHD and its underlying anatomy. It is important to emphasize that optimal visualization of individual ventricular septal defects requires the use of appropriate echocardiographic views [63]. On chest radiography, tetralogy of Fallot (ToF) is classically characterized by a boot-shaped cardiac silhouette [75]. Cardiac magnetic resonance imaging (CMR) and cardiac computed tomography (CCT) with three-dimensional (3D) reconstruction represent the gold-standard imaging modalities for the diagnosis and anatomical assessment of coarctation of the aorta (CA) [80].

Patients with CHD frequently exhibit chromosomal abnormalities or aneuploidies (8–12%), copy number variations (3–25%), and pathogenic single-gene variants (3–5%), while the reported incidence of de novo mutations is approximately 8%. [85,87]. Complex CHD frequently occur as part of genetic syndromes, including Turner syndrome, DiGeorge syndrome (22q11.2 deletion syndrome), Down syndrome, and Williams–Beuren syndrome [26,73]. Genes with established roles in the pathogenesis of CHD predominantly encode transcription factors, signaling molecules, and structural proteins that are essential for cardiac development, morphogenesis, and normal cardiac function [Table 1]. Individuals with a family history of CHD in a first-degree relative have a 3- to 80-fold higher risk of CHD than the general population. In familial CHD, pathogenic variants in specific genes, such as NKX2-5, which is strongly associated with atrial ASD and atrioventricular (AV) block, are associated with poorer clinical outcomes and an increased risk of sudden cardiac death [89].

Late diagnosis of CHDs is characteristic of underdeveloped and developing countries, primarily due to limited healthcare resources and the smaller number and availability of specialized centers where such diagnoses can be established. At the same time, these populations also have the least access to complex and costly forms of surgical treatment [96]. Late presentation in the natural history of CHD consequently leads to the progression of pathophysiological processes and the development of unfavorable hemodynamic conditions in certain types of CHD [96,97,98]. In infants and children with undiagnosed complex CHDs, such as TGA without a significant ASD or VSD, early circulatory collapse and multiorgan failure may occur. In patients with Eisenmenger syndrome, for example, secondary polycythemia and coagulation disorders may develop, while untreated valvular disease may predispose to infective endocarditis. Consequently, late correction of CHD is associated with a high perioperative risk, an increased incidence of postoperative complications, and higher mortality [96,97,99]. As expected, patients with late-diagnosed and untreated congenital heart disease (CHD) have a reduced life expectancy compared with the general population. The natural clinical course was followed in 20 patients with ostium secundum ASD and established significant pulmonary hypertension. All patients had a delayed diagnosis of CHD. The ASD was detected at a mean age of 39 years, although some patients had been symptomatic since adolescence. Notably, 46% of the patients died from cardiac causes at a mean age of 51 years [100]. Another study investigated a cohort of 79 patients with VSD diagnosed during adulthood, at a mean age of 34 years. A small proportion of patients (n = 12) underwent surgical repair, whereas the remaining patients received medical treatment. Overall, the 10-year survival rate was 76% and was associated with the patients’ clinical status at the beginning of follow-up, including NYHA functional class, the presence of moderate-to-severe pulmonary hypertension, and documented cardiac chamber dilatation [101]. In prior study of patients with late-detected and untreated coarctation of the aorta, a high mortality rate was documented during the natural course of the disease, with mortality reaching 25% within the first two decades of life, 50% by the age of 32 years, 75% by the age of 46 years, and as high as 90% by the age of 58 years [102]. A systematic review of the literature involving more than 1000 patients with untreated Eisenmenger syndrome demonstrated a very high 10-year mortality rate, reaching 30–40% [103].

Given the high prevalence of comorbidities and the substantial mortality among untreated adults with late-diagnosed CHD, improving the early detection of CHD is essential to enable timely diagnosis and appropriate treatment. The previously described higher incidence of late-detected and undiagnosed CHD in adulthood in low-resource countries and regions, compared with developed rural areas, is likely attributable to limited diagnostic capabilities in these settings. Although echocardiography is considered the gold standard for the detection of CHD in newborns, its sensitivity and specificity still do not consistently reach the desired levels. Fetal echocardiography has also proven to be a useful diagnostic tool; although its sensitivity is moderate, its high specificity allows for reliable exclusion of CHD in cases with negative findings. Finally, pulse oximetry represents the simplest and most accessible diagnostic method for the detection of complex CHD [104]. The limited sensitivity and specificity of transthoracic echocardiography (TTE) are attributable not only to differences in the availability and quality of echocardiographic equipment but also to its substantial dependence on the expertise and diagnostic capabilities of the diagnostician. Therefore, an important question is how diagnostic methods can be standardized and made more widely accessible on a global scale, and to what extent artificial intelligence (AI)-based tools may contribute to addressing these challenges. A study published as early as 2022 demonstrated the impact of artificial intelligence (AI) technology, specifically a deep learning-based explainable representation in the form of a “graph chart diagram” to support fetal cardiac ultrasound screening, on improving CHD detection across all levels of diagnostic expertise, from specialists to residents. However, diagnostic performance still differed according to the level of training and experience of the diagnostician [105]. Computer-assisted auscultation could also serve as a screening tool for CHD detection, given the demonstrated 98% sensitivity, 91% specificity, and 97% accuracy of remote auscultation in detecting abnormal heart sounds [106]. As expected, AI technology could be particularly useful in detecting small cardiac defects that might otherwise be missed by conventional diagnostic methods, such as small ASDs or partial anomalous pulmonary venous return.

## 8. Conclusions

CHDs may remain undiagnosed until adulthood, whether they involve minor, asymptomatic defects or previously unrecognized variants of complex CHDs with an associated shunt or a more favorable anatomical configuration that permits survival into adulthood. The initial presentation of CHD in adulthood is most often recognized only after the development of complications, such as HF, arrhythmias, right-sided cardiac chamber dilatation, or pulmonary hypertension. Previously unrecognized CHD in adults should be suspected in the presence of symptoms such as dyspnea, palpitations, chest pain or discomfort, and syncope, as well as signs of heart failure. Suspicion should be particularly high in patients presenting with specific electrocardiographic abnormalities, including tall P waves, PR interval prolongation, right bundle branch block (RBBB), and fragmented QRS complexes. In younger patients, particular attention should be paid to findings on the initial echocardiographic examination, including heart failure with reduced ejection fraction (HFrEF), left atrial dilatation, dilatation of the right-sided cardiac chambers, and pulmonary hypertension. In such cases, a detailed transthoracic echocardiographic (TTE) assessment is warranted and should be complemented, when indicated, by additional imaging modalities, including 3D TTE, 3D TEE, CT, CMR, and scintigraphy. Patients with CHD frequently exhibit chromosomal abnormalities or aneuploidies; however, a high incidence of de novo mutations has also been reported. Certain forms of CHD have a substantial genetic component, as exemplified by bicuspid aortic valve (BAV), and therefore genetic screening of family members should be considered when appropriate. In adult patients presenting with newly diagnosed CHD, accurate recognition of clinical symptoms and completion of an appropriate diagnostic workup are essential for establishing the diagnosis and initiating timely treatment. Future research should focus on the development and validation of appropriate screening strategies for the early detection of congenital heart diseases (CHDs).

## Figures and Tables

**Table 1:** Genes with evidence for CHD (ClinGen).

Gene ^1^	Disease	Inheritance Pattern
*GATA4*	structural congenital heart disease, multiple types—GATA4 (ASD, ToF, VSD)	AD
*GATA6*	GATA6-related congenital heart disease with or without pancreatic agenesis or neonatal diabetes (ASD, VSD, BAV, PDA, TGA, ToF, Conotruncal heart malformations)	AD
*ISL1*	congenital heart disease	AD
*MYH6*	MYH-6-related congenital heart defects (ASD, atrioventricular septal defects, conotruncal defects, tricuspid atresia and left sided obstructive lesions)	AD
*NKX2-5*	NKX2.5-related congenital, conduction and myopathic heart disease (ASD, VSD, conotruncal heart malformations, Hypoplastic left heart syndrome)	AD
*NODAL*	congenital heart disease with heterotaxy syndrome	AD
*NR2F2*	NR2F2-related multiple congenital anomalies/dysmorphic syndrome	AD
*PLD1*	PLD1-related congenital heart disease	AR
*RBM10*	TARP syndrome	XL
*SMAD2*	congenital heart disease (ASD, VSD, dextrocardia, atrial isomerism, double-outlet right ventricle, unbalanced complete atrioventricular canal, unbalanced right-dominant complete atrioventricular canal, pulmonary stenosis, pulmonary atresia, mitral atresia and hypoplastic left ventricle)	AD
*TAB2*	congenital heart defects, multiple types, 2 (ASD, BAV, valvular dysplasia, valvular stenosis, VSD)	AD
*TFAP2B*	TFAP2B-related congenital heart disease spectrum disorder (PDA)	AD
*ZIC3*	congenital heart disease with heterotaxy syndrome	XL
*RBFOX2*	congenital heart disease (aortic stenosis/atresia, ASD, double outlet right ventricle, hypoplastic aortic arch, and hypoplastic left ventricle)	AD
*CITED2*	congenital heart disease	AD
*ETS1*	congenital heart disease (hypoplastic left heart syndrome)	AD
*FGF8*	congenital heart disease	AD
*HAND1*	congenital heart disease (double-outlet right ventricle with VSD, ToF, VSD)	AD
*HAND2*	HAND2-related congenital heart defect	AD
*KLF13*	congenital heart disease	AD
*MESP1*	congenital heart disease	AD
*ROBO4*	aortic valve disease 3 (aortic root aneurysm, ascending aortic aneurysm, ASD)	AD
*TBX20*	congenital heart disease	AD

^1^ Gene names highlighted in green indicate genes with **definitive evidence** for an association with CHD. Gene names highlighted in blue indicate genes with **strong evidence**, whereas those highlighted in orange indicate genes with **moderate evidence** for an association with CHD.

## Data Availability

No new data were created or analyzed in this study. Data sharing is not applicable to this article.

## Abbreviations

The following abbreviations are used in this manuscript:

CHD	Congenital heart diseases
ASD	Atrial septal defect
PDA	Patent ductus arteriosus
CA	Coarctation of the aorta
ToF	Tetralogy of Fallot
ccTGA	Congenitally corrected transposition of the great arteries
MV	Mitral valve
BAV	Bicuspid aortic valve
RV	Right ventricle
PV	Pulmonary valve
VSD	Ventricular septal defect
TGA	Transposition of the great arteries
CAD	Coronary artery disease
HF	Heart failure
NYHA	New York Heart Association
RVOT	Right ventricular outflow tract
HFpEF	Heart failure with preserved ejection fraction
HFrEF	Heart failure with reduced ejection fraction
d-TGA	Dextro-transposition of the great arteries
AV	Atrioventricular
AVRT	Atrioventricular reciprocating tachycardia
VT	Ventricular tachycardia
RBBB	Right bundle branch block
PFO	Patent foramen ovale
ECG	Electrocardiography
WPW	Wolff–Parkinson–White syndrome
2D	Two-dimensional
3D	Three-dimensional
4D	Four-dimensional
GLS	Global longitudinal strain
LA	Left atrium
TEE	Transesophageal echocardiography
CMR	Cardiac magnetic resonance imaging
CCT	Cardiac computed tomography
PVR	Pulmonary vascular resistance
AR	Aortic regurgitation
AVSD	Atrioventricular septal defects
TTE	Transthoracic echocardiography
IVS	Interventricular septum
sPAP	Systolic pulmonary artery pressure
DCRV	Double-chambered right ventricle
BSA	Body surface area
RVEDV	Right ventricular end-diastolic volume
RVESV	Right ventricular end-systolic volume
DORV	Double-outlet right ventricle
LVOT	Left ventricular outflow tract
LVNC	Left ventricular noncompaction
